# Postoperative Pain After Root Canal Preparation With Different Instruments in Primary Teeth: A Systematic Review and Meta‐Analysis

**DOI:** 10.1002/cre2.70180

**Published:** 2025-07-24

**Authors:** Kavalipurapu Venkata Teja, Kaligotla Apoorva Vasundhara, Gianrico Spagnuolo, Mariangela Cernera, Niccolò Giuseppe Armogida, Flavia Iaculli, Carlo Rengo

**Affiliations:** ^1^ Department of Conservative Dentistry & Endodontics Mamata Institute of Dental Sciences Hyderabad Telangana India; ^2^ Department of Prosthodontics, Saveetha Dental College and Hospitals – Saveetha Institute of Medical and Technical Sciences Saveetha University Chennai India; ^3^ Department of Neurosciences, Reproductive and Odontostomatological Sciences University of Naples Federico II Naples Italy; ^4^ Department of Oral and Maxillofacial Sciences Sapienza University of Rome Rome Italy

**Keywords:** endodontic treatment, mechanical instrumentation, pain, primary teeth, pulpectomy, root canal preparation

## Abstract

**Objective:**

This systematic review and meta‐analysis aimed to evaluate the effectiveness of different instrumentation systems in reducing postoperative pain following root canal preparation in primary teeth.

**Material and Methods:**

The present study was conducted in accordance with PRISMA guidelines and registered on PROSPERO (CRD42020135904). The review aimed to determine whether there is a difference in postoperative pain incidence using various instrumentation systems (manual and mechanical) for root canal preparation of primary teeth during pulpectomy. An extensive database search was performed using specific MeSH terms to include clinical studies up to November 2024. Based on eligibility criteria, the selected articles were subjected to quality assessment and the risk of bias was conducted using the Cochrane Risk of Bias (RoB 2) tool. In addition, meta‐analyses were conducted on homogeneous studies.

**Results:**

A total of 11 studies were included for qualitative assessments, and 7 studies underwent quantitative analysis. The results review indicated that mechanical instrumentation systems yielded better overall pain reduction compared to manual systems. The meta‐analysis further demonstrated statistically significant pain reduction at 6 (*p* < 0.01, 95% CI: 1.46) and 12 h (*p* < 0.01, 95% CI: 2.15). However, no notable pain reduction or significance were observed at other time points (*p* = 0.41, 95% CI: 1.66; *p* = 0.23, 95% CI: 1.67; *p* = 0.61, 95% CI: 1.25). The overall risk of bias was low for the included studies.

**Conclusion:**

Rotary NiTi instrumentation systems were superior in reducing Postoperative pain incidence in primary teeth undergoing pulpectomy.

**Clinical Relevance:**

Mechanical instrumentation is not only advantageous in decreasing overall treatment time, but also in reducing pain incidence after pulpectomy, which nowadays represents an important and widely used procedure to preserve primary teeth.

## 1 Introduction

The preservation of primary teeth until the eruption of permanent successors is of paramount importance for the space maintenance, good development of the oral apparatus, and correct masticatory function (Cleghorn et al. 2010). Irreversible pulp inflammation and involvement of periapical tissue of primary teeth in most cases require pulpectomy to retain the involved tooth until exfoliation and allow normal growth pattern of permanent element (Li et al. 2023). Although endodontic treatment is a widely accepted procedure even in primary dental elements, it's still considered challenging due to the wide range of anatomical variations of the root canal system of primary teeth (Ahmed et al. 2020). Moreover, the success of the therapy over time depends on several factors as proper removal of infected tissue, adequate preparation and disinfection of the root canals, and effective obturation and sealing (Girish Babu et al. 2024). Compared to traditional methods using manual K‐files and H‐files, modern rotary and reciprocating systems are mostly employed to facilitate root shaping (Manchanda et al. 2020; Schachter et al. 2023). Moreover, the introduction of nickel‐titanium (NiTi) files has significantly shortened treatment times in pediatric endodontics (Shetty et al. 2023). Even though some clinicians limit the use of rotary files due to concerns about iatrogenic errors or incomplete pulp tissue removal (Nisar et al. 2024), evidence‐based studies have shown that rotary NiTi files reduce overall treatment time, particularly instrumentation time, while promoting conservative dentin removal and more consistent conical root canal preparations (Panchal, Jeevanandan, and Erulappan 2019). The reciprocating files, as an alternative to the rotary ones, offer enhanced safety during instrumentation, contributing to more efficient canal cleaning (Marques et al. 2023).

It's been widely demonstrated within scientific literature that rotary and reciprocating systems are effective in post‐endodontic pain reduction in the case of permanent teeth (Sun et al. 2018; Martins et al. 2019; Rahbani Nobar et al. 2021); however, clinical data regarding their impact on postoperative pain in primary teeth remain limited (Manchanda et al. 2020; Lakshmanan et al. 2021). Post‐instrumentation pain in pediatric patients typically ranges from mild discomfort to moderate or severe pain, lasting from a few hours to several days (Lakshmanan et al. 2021). In general, posttreatment pain is due to several factors including patient‐related, operator‐related, and treatment protocol‐related factors (Arias et al. 2013). During root canal debridement, extruded dentin debris, combined with microbial agents and chemical disinfectants, can provoke an acute inflammatory response in the periapical tissue, leading to post‐endodontic pain (Sun et al. 2018; Ng et al. 2004). In vitro studies demonstrated that some degree of dentin debris extrusion is inevitable with any instrument design (Suresh et al. 2023).

Recent clinical trials have assessed the effects of different instrumentation systems on postoperative pain in pediatric patients (Asokan et al. 2021; Morankar et al. 2020); however, few data are available to reach a univocal agreement in terms of pain reduction. Therefore, the aim of the present systematic review and meta‐analysis was to evaluate the incidence of postoperative pain following root canal preparation in primary teeth using different manual or mechanical instrumentations.

## 2 Materials and Methods

The present systematic review was conducted following the PRISMA guidelines (Page et al. 2021), and the protocol was registered on PROSPERO (CRD42020135904). The review aimed to answer the following question: “Is there a difference in postoperative pain incidence when using different instrumentation systems during root canal preparation of primary teeth undergoing pulpectomy?” The PICO framework was as follows:

**Population:** Subjects with primary teeth underwent pulpectomy.

**Intervention:** Mechanical root canal preparation with different NiTi instrumentation systems.

**Comparison:** Manual preparation of the root canal system.

**Outcome:** Postoperative pain incidence.

### 2.1 Search Strategy

A comprehensive literature search was performed through multiple electronic databases including PubMed, Scopus, Embase, Google Scholar, Web of Science, and Cochrane up to November 2024. Relevant MeSH terms were used as follows: ((((Primary teeth) OR (Deciduous teeth)) AND (((((Root canal preparation) OR (Endodontic treatment)) OR (Root canal therapy)) OR (Pulp therapy)) OR (Pulpectomy))) AND (((Manual instrumentation) OR (Rotary instrumentation)) OR (Reciprocating instrumentation))) AND (((Postoperative pain) OR (Posttreatment pain)) OR (Post‐endodontic pain)).

In addition, reference lists of the evaluated articles were manually searched to identify other studies for potential inclusion.

### 2.2 Eligibility Criteria

The following inclusion and exclusion criteria were used to select the studies.

#### 2.2.1 Inclusion Criteria

– Studies published in Peer‐reviewed Journals.

– Studies published in English language.

– Studies reporting clinical trials: randomized clinical trials, prospective comparative clinical trials;‐ Studies reporting on root canal preparation of primary teeth with different instrumentation systems (hand files, rotary, or reciprocating instrumentation) and evaluating postoperative pain incidence.

#### 2.2.2 Exclusion Criteria

– Laboratory‐based studies and ex vivo studies.

– Studies on animal samples.

– Trials involving permanent teeth.

– Case series, case report, reviews and retrospective studies.

– Gray literature.

### 2.3 Study Selection

Screening and selection of papers were performed by two independent reviewing authors (K.V.T. and K.A.V.) following the eligibility criteria. After the removal of duplicates, studies were assessed by title and abstract. Then, potentially relevant full texts were retrieved for reading and data extraction. Any discrepancies in the study selection process were solved by a third reviewer (G.S.).

### 2.4 Data Extraction and Qualitative Analysis

The collected data were independently reported by the two reviewers in Excel sheets for further analysis. The following information for each included study was described: authors, year, study design, sample size, age, tooth type, pulpal/periapical condition, instrumentation technique, number of treatment sessions, obturation material used, irrigants or intracanal medicaments, pain assessment scale, prescription of posttreatment analgesics, evolution period, comparison between experimental groups.

Qualitative assessments were conducted on the selected articles using a revised Cochrane risk‐of‐bias tool for randomized trials (RoB 2) (Sterne et al. 2019) by means of Review Manager 5.4 software (RevMan, The Nordic Cochrane Centre, Copenhagen, Denmark). Based on the 5 assessed domains, the selected studies were categorized as having low risk of bias (all domains were satisfied), some concerns (in at least one domain), or high risk of bias (high risk in at least one domain or some concerns for multiple domains).

### 2.5 Quantitative Synthesis

Meta‐analyses were conducted only on homogeneous studies using Review Manager 5.4 software (RevMan, The Nordic Cochrane Centre, Copenhagen, Denmark). The risk ratio (RR) was calculated to compare the pain rate between the manual instrumentation group and the mechanical instrumentation group, and the results were reported with 95% confidence intervals (CIs). Analysis of overall pain incidence that pooled together all subjects who experienced pain after manual or mechanical instrumentation was performed. In addition, subgroup analyses were carried out based on the time intervals after pulpectomy (6, 12, 24, 48, and 72 h after treatment). Statistical heterogeneity among studies was assessed using the chi‐squared test and the Higgins index (I^2^). The I^2^ statistic represents the percentage of diversity in effect estimates due to heterogeneity, rather than sampling error. Pooled estimates were calculated using the Mantel–Haenszel fixed‐effects model of analysis if I^2^ ≤ 50%; otherwise, a random‐effects model of analysis was applied. All results were expressed as RRs with 95% CIs and shown in forest plots.

## 3 Results

**Figure 1:**
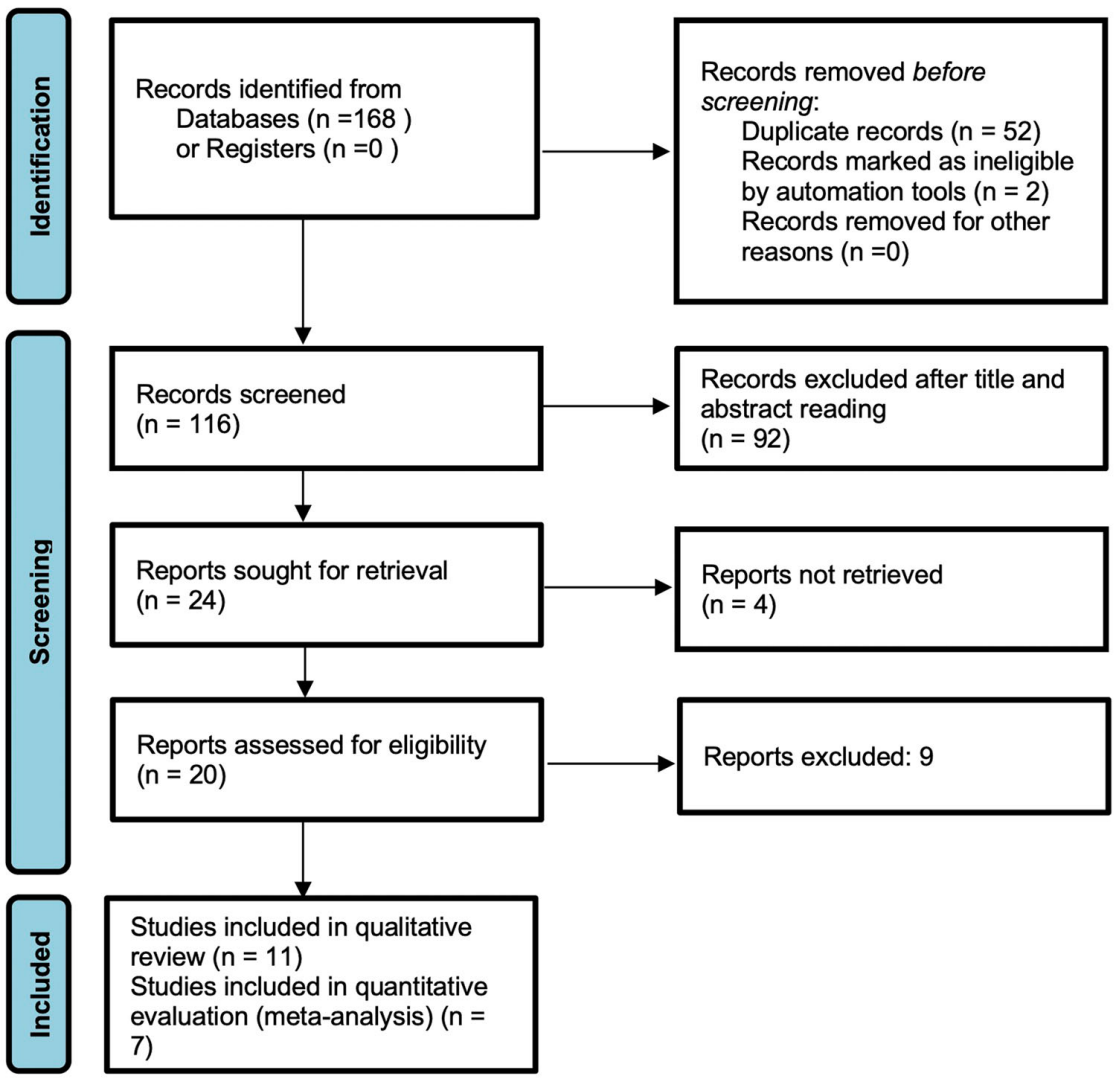
PRISMA flow chart describing the search strategy.

The search strategy identified a total of 168 studies. Following duplicates removal, 116 papers were screened for title and abstract reading, and 20 studies underwent full‐text reading. According to eligibility criteria, 11 papers were included in the present systematic review and processed for qualitative analysis (Marques et al. 2023; Hadwa et al. 2023; Thakur et al. 2023; Panchal, Jeevanandan, and Subramanian 2019; Jeevanandan et al. 2020; Topçuoğlu et al. 2017; Barasuol et al. 2021; Tyagi et al. 2021; Moudgalya et al. 2024; Divya et al. 2019; Bohidar et al. 2024); in addition, 7 studies (Marques et al. 2023; Hadwa et al. 2023; Thakur et al. 2023; Panchal, Jeevanandan, and Subramanian 2019; Jeevanandan et al. 2020; Topçuoğlu et al. 2017; Divya et al. 2019) underwent quantitative evaluation (meta‐analysis) (Figure 1). Characteristics and outcomes of included papers are summarized in Tables 1 and 2.

**Table 1:** Characteristics of the included studies.

Study	Type of study	Sample size	Age	Teeth type	Pulpal/periapical condition	Groups' distribution	Instrumentation technique	Number of treatment sessions/obtuartion material	Irrigants or intracanal medicaments	Pain assessment scale	Posttreatment analgesic prescribed
Marques et al. (2023)	Randomized controlled trial	151 teeth	3–9 years	Primary molars	Irreversible pulpitis or apical periodontitis	Group I: Manual K‐files Group II: Kedo‐S Square rotary files Group III: WaveOne Gold files	Crown‐down technique	Single visit/Obturation: iodoform‐calcium hydroxide (Vitapex)	1% NaOCl during instrumentation + final rinse EDTA	Wong‐Baker pain rating scale	Unclear
Hadwa et al. (2023)	Randomized controlled trial	60 teeth	4–7 years	Mandibular primary second molars	Non‐vital pulp	Group I: Kedo‐S Square rotary files Group II: Fanta AF^_^ rotary system Group III: Manual K‐files	Rotary files: crown down technique; Manual files: Quarter‐turn‐pull technique	Single visit/Obturation: iodoform‐calcium hydroxide (Metapex)	17% EDTA before instrumentation, 1% NaOCl in between files + final flush with normal saline	Four‐point pain intensity scale	Unclear
Thakur et al. (2023)	Randomized controlled trial	75 teeth	4–9 years	Mandibular primary molars	Pulpitis/absence of periapical lesions	Group I: XP Endo shaper files Group II: Kedo SG Blue files Group III: Manual K‐files	XP Endo shaper: continuous rotary movement; Rotary files: crown down technique; Manual files: quarter‐turn‐pull technique	Single visit/Obturation: iodoform‐calcium hydroxide (Metapex)	2.5% NaOCl during instrumentation + final rinse of 17% EDTA + final flush normal saline	Wong‐Baker pain rating scale	Ibuprofen or paracetamol
Panchal, Jeevanandan, and Subramanian (2019)	Randomized controlled trial	75 teeth	4–6 years	Posterior primary teeth	Pulpal necrosis	Group I: Manual K‐file Group II: Manual H‐ file Group III: Kedo S files	Rotary files: crown down technique; Manual K‐files: quarter‐turn‐pull technique; Manual H‐files: retraction technique	Single visit/Obturation: iodoform‐calcium hydroxide (Metapex)	Normal saline solution	Modified Wong‐Baker pain rating scale	Ibuprofen or paracetamol
Jeevanandan et al. (2020)	Randomized controlled trial	60 patients	6–8 years	Maxillary primary teeth	Asymptomatic irreversible pulpitis/absence of periapical lesion	Group I: Manual NiTi K‐flex files Group II: Reciprocating NiTi flex K‐files Group III: Kedo S rotary files	Rotary files: crown down technique; Manual K‐files: quarter‐turn‐pull technique; Manual H‐files: retraction technique	Single visit/Obturation: iodoform‐zinc‐oxide (Endoflas)	1% NaOCl + EDTA Gel during instrumentation + final rinse of 2% CHX + final flush normal saline	Four‐point pain intensity scale	Ibuprofen or paracetamol
Topçuoğlu et al. (2017)	Randomized controlled trial	106 patients	6–8 years	Maxillary primary molars	Pulpal necrosis/absence of periapical lesion	Group I: Manual hand K‐file Group II: Revo‐S rotary files	Rotary files: crown down technique, Manual K‐ files: quarter‐turn‐pull technique	Single visit/Obturation: zinc‐oxide eugenol paste	1% NaOCl between each file.	Four‐point pain intensity scale	Ibuprofen or paracetamol
Barasuol et al. (2021)	Randomized controlled trial	88 patients	4–9 years	Primary molars	Pulp necrosis or irreversible pulpitis/with or without periapical lesion	Group I: Manual hand K‐file Group II: ProDesign Logic rotary files	Rotary files: crown down technique; Manual K‐files: crown down rotation and traction technique	Single visit/Obturation: zinc‐oxide eugenol paste	2.5% NaOCl during instrumentation + final rinse of 17% EDTA and 2.5% NaOCl.	Faces Pain Scale Revised	Unclear
Tyagi et al. (2021)	Randomized controlled trial	75 teeth	4–8 years	Primary molars	Irreversible pulpits, pulpal necrosis, apical periodontitis	Group I: Manual hand K‐file NiTi flex Group II: ProAF baby gold rotary files Group III: WaveOne Gold reciprocating files	Rotary and reciprocating file: crown down technique; Manual K‐files NiTi flex: quarter‐turn‐pull technique	Single visit/Obturation: iodoform‐calcium hydroxide (Metapex)	1% NaOCl followed by normal saline	Four‐point pain intensity scale	Unclear
Divya et al. (2019)	Randomized controlled trial	45 teeth	6–8 years	Mandibular primary molars	Asymptomatic irreversible pulpitis	Group I: Manual K‐files Group II: Kedo‐S rotary files Group III: K3 rotary files	Rotary files: crown down technique; Manual K‐files: quarter‐turn‐pull technique	Single visit/Obturation: iodoform‐calcium hydroxide (Metapex)	3% NaOCl during instrumentation + normal saline	Four‐point pain intensity scale	Unclear
Moudgalya et al. (2024)	Randomized controlled trial	75 patients	5–9 years	Primary molars	Pulpitis or apical periodontitis	Group I: Kedo S rotary files Group II: Rotary K‐flex files Group III: Hand K‐file or H‐files	Crown down technique	Two visits/Unclear medication/Obturation: iodoform‐calcium hydroxide (Metapex)	0.2% CHX and normal saline alternatively in Groups II and III. 17% EDTA gel during instrumentation in Group I.	Unclear	Unclear
Bohidar et al. (2024)	Clinical trial with random allocation	36 teeth	4–8 years	Primary molars	Unclear	Group I: Manual hand K‐file Group II: Kedo S rotary files	Rotary files: crown down technique; Manual K‐files: balanced force technique	Single visit/Obturation: iodoform‐calcium hydroxide (Metapex)	3% NaOCl during instrumentation + normal saline	Four‐point pain intensity scale	Unclear

**Table 2:** Outcomes reported by the included studies.

Study	Groups	Evaluation period	Preoperative pain	Pain assessments	Analgesic intake
Marques et al. (2023)	Group I: Manual K‐files Group I: Kedo‐S Square rotary files Group III: Wave One Gold files	48 h	Not reported	Manual Hand K‐files = WaveOne Gold files	Manual Hand K‐ files = WaveOne Gold files
Hadwa et al. (2023)	Group I: Kedo‐S Square rotary files Group II: Fanta AF^_^ rotary system Group III: Manual K‐files	6, 12, 24, 48 h	Not reported	Kedo‐S Square rotary files > Fanta AF^_^ rotary system > Manual K‐files	Not assessed
Thakur et al. (2023)	Group I: XP Endo shaper files Group II: Kedo SG Blue files Group III: Manual K‐files	6, 12, 24, 48, and 72 h	Reported	XP‐ Endo shaper > Kedo‐S Square rotary files > Manual K‐files	Not assessed
Panchal, Jeevanandan, and Subramanian (2019)	Group I: Manual K‐file Group II: Manual H‐ file Group III: Kedo S files	6, 12, 24, 48 and 72 h	Not reported	Kedo‐S Square rotary files > Manual K‐files > Manual H‐files	Not assessed
Jeevanandan et al. (2020)	Group I: Manual NiTi K‐flex files Group II: Reciprocating NiTi flex K‐files Group III: Kedo S rotary files	6, 12, 24, 48, and 72 h	Reported	Kedo‐S Square rotary files > Reciprocating NiTi K‐flex files > Manual H‐files	Not assessed
Topçuoğlu et al. (2017)	Group I: Manual hand K‐file Group II: Revo‐S rotary files	12, 24, 48, 72 h, and one week	Not reported	Revo‐S rotary files > Manual K‐files	Not assessed
Barasuol et al. (2021)	Group I: Manual hand K‐file Group II: ProDesign Logic rotary files	6 and 72 h (data was pooled together and dichotomized)	Not reported	Manual hand K‐file = ProDesign Logic rotary files	Manual hand K‐file = ProDesign Logic rotary files
Tyagi et al. (2021)	Group I: Manual hand K‐file NiTi flex Group II: ProAF baby gold rotary files Group III: WaveOne Gold reciprocating files	6, 24, 72 h and 1 week	Reported	At 6 h: WaveOne gold reciprocating files > ProAF baby gold rotary files > Manual hand K‐file NiTi flex At other time intervals: WaveOne Gold reciprocating files = ProAF baby gold rotary files = Manual hand K‐file NiTi flex	Not assessed
Divya et al. (2019)	Group I: Manual K‐files Group II: Kedo‐S rotary files Group III: K3 rotary files	6, 12, 24, 48, and 72 h	Not reported	Manual K‐files = Kedo‐S rotary files = K3 rotary files	Not assessed
Moudgalya et al. (2024)	Group I: Kedo S rotary files Group II: Rotary K‐flex files Group III: Hand K‐file or H‐files	3‐day time intervals (unclear on the actual time intervals assessed)	Not reported	Kedo S rotary files > Rotary K‐flex files > Hand K‐file or H‐files	Not assessed
Bohidar et al. (2024)	Group I: Manual hand K‐file Group II: Kedo S rotary files	6, 12, 24, 48, and 72 h	Reported	Kedo S files > Manual hand K‐files	Not assessed

A total of 10 included studies were randomized controlled trials (Marques et al. 2023; Hadwa et al. 2023; Thakur et al. 2023; Panchal, Jeevanandan, and Subramanian 2019; Jeevanandan et al. 2020; Topçuoğlu et al. 2017; Barasuol et al. 2021; Tyagi et al. 2021; Moudgalya et al. 2024; Divya et al. 2019), while 1 study was a clinical trial (Bohidar et al. 2024). The age range of the study participants was 3–9 years. Regarding evaluated primary teeth, 6 studies included unspecified primary molars (Marques et al. 2023; Panchal, Jeevanandan, and Subramanian 2019; Barasuol et al. 2021; Tyagi et al. 2021; Moudgalya et al. 2024; Bohidar et al. 2024), 3 studies focused on mandibular primary molars (Hadwa et al. 2023; Thakur et al. 2023; Divya et al. 2019), and 2 studies specifically analyzed maxillary primary posterior teeth (Jeevanandan et al. 2020; Topçuoğlu et al. 2017). In 10 out 11 studies, the entire pulpectomy procedure was completed in a single visit (Marques et al. 2023; Hadwa et al. 2023; Thakur et al. 2023; Panchal, Jeevanandan, and Subramanian 2019; Jeevanandan et al. 2020; Topçuoğlu et al. 2017; Barasuol et al. 2021; Tyagi et al. 2021; Divya et al. 2019; Bohidar et al. 2024), while in 1 article a two‐visit protocol was adopted (Moudgalya et al. 2024). Regarding postoperative pain levels evaluation after pulpectomy, observational time intervals were different. Indeed, 5 studies analyzed pain levels from 6 h to 3 days postoperatively (Thakur et al. 2023; Panchal, Jeevanandan, and Subramanian 2019; Jeevanandan et al. 2020; Topçuoğlu et al. 2017; Divya et al. 2019) and 2 studies assessed pain only up to 2 days postoperatively (Marques et al. 2023; Hadwa et al. 2023). Only 2 papers reported outcomes after 1 week (Topçuoğlu et al. 2017; Tyagi et al. 2021). Finally, 2 studies reported pooled and dichotomized 3‐day pain evaluations (Barasuol et al. 2021; Moudgalya et al. 2024), although the specific time intervals used for evaluations were unclear.

Sample sizes varied across the included studies. Precisely, some of them considered the number of patients (Moudgalya et al. 2024), while others evaluated the number of teeth underwent pulpectomy (Marques et al. 2023; Hadwa et al. 2023; Tyagi et al. 2021; Divya et al. 2019; Bohidar et al. 2024). The statistical power of the included studies ranged from 80% (Marques et al. 2023; Hadwa et al. 2023; Thakur et al. 2023; Jeevanandan et al. 2020; Tyagi et al. 2021; Moudgalya et al. 2024) to 95% (Panchal, Jeevanandan, and Subramanian 2019; Divya et al. 2019). The study by Bohidar et al. (2024) did not provide a sample size calculation.

Regarding preoperative conditions, 4 studies enrolled only teeth with pulpal necrosis (Hadwa et al. 2023; Panchal, Jeevanandan, and Subramanian 2019; Topçuoğlu et al. 2017; Barasuol et al. 2021; Tyagi et al. 2021), while others also included cases of pulpitis or apical periodontitis (Marques et al. 2023; Thakur et al. 2023; Jeevanandan et al. 2020; Divya et al. 2019; Moudgalya et al. 2024); the diagnostic inclusion criteria were unclear in one study (Bohidar et al. 2024).

Rotary canal instrumentation was obtained by Kedo‐S rotary files (Marques et al. 2023; Hadwa et al. 2023; Panchal, Jeevanandan, and Subramanian 2019; Jeevanandan et al. 2020; Divya et al. 2019; Moudgalya et al. 2024; Bohidar et al. 2024), Fanta files (Hadwa et al. 2023), Revo‐S (Topçuoğlu et al. 2017), Rotary K‐flex files (Moudgalya et al. 2024), K3 Rotary files (Divya et al. 2019), ProDesign Logic rotary files (Barasuol et al. 2021), ProAF Baby Gold rotary files (Tyagi et al. 2021), and XP Endo Shaper file systems (Thakur et al. 2023). The reciprocating motion was used in 3 out 11 studies such as WaveOne Gold file system (Marques et al. 2023; Tyagi et al. 2021) and NiTi Flex file system (Jeevanandan et al. 2020). On the other hand, manual root canal preparation was carried out with K‐files (Marques et al. 2023; Hadwa et al. 2023; Thakur et al. 2023; Topçuoğlu et al. 2017; Barasuol et al. 2021; Divya et al. 2019; Moudgalya et al. 2024), K‐Niti Flex files (Jeevanandan et al. 2020; Tyagi et al. 2021), or H‐files (Panchal, Jeevanandan, and Subramanian 2019; Moudgalya et al. 2024).

Hand K‐files employed the quarter‐turn and pull motion, while H‐files employed the retraction and pull technique; only Bohidar et al. (2024) used a balanced force technique with manual K‐files. All studies using rotary or reciprocating file systems employed a crown‐down technique.

Irrigation agents varied among the included studies. 1% NaOCl was the most commonly used primary irrigant (Marques et al. 2023; Hadwa et al. 2023; Jeevanandan et al. 2020; Topçuoğlu et al. 2017; Tyagi et al. 2021), followed by 3% NaOCl (Divya et al. 2019; Bohidar et al. 2024) and 2.5% NaOCl without dilution (Thakur et al. 2023; Barasuol et al. 2021). Two studies (Jeevanandan et al. 2020; Moudgalya et al. 2024) reported the use of 2% CHX, either as an irrigant or a final flush. Final rinse of 17% EDTA was used in 5 studies (Marques et al. 2023; Hadwa et al. 2023; Thakur et al. 2023; Barasuol et al. 2021; Moudgalya et al. 2024), while in 2 papers, EDTA was applied as gel during instrumentation (Jeevanandan et al. 2020; Moudgalya et al. 2024). Only one study used saline solution as irrigant from the initial instrumentation phase up to final shaping (Panchal, Jeevanandan, and Subramanian 2019).

Regarding obturation materials, Ca(OH)₂ with iodoform (Metapex or Vitapex) was the most commonly used agent (Marques et al. 2023; Hadwa et al. 2023; Thakur et al. 2023; Panchal, Jeevanandan, and Subramanian 2019; Tyagi et al. 2021; Moudgalya et al. 2024; Divya et al. 2019; Bohidar et al. 2024). Combinations of zinc oxide and iodoform (Endoflas) was used in 1 study (Jeevanandan et al. 2020), while 2 studies applied zinc oxide eugenol as final obturation (Topçuoğlu et al. 2017; Barasuol et al. 2021).

Pain rating scales used to evaluate pain level were different among studies. Specifically, 7 papers used a four‐point pain intensity scale for pain analysis (Hadwa et al. 2023; Jeevanandan et al. 2020; Topçuoğlu et al. 2017; Barasuol et al. 2021; Tyagi et al. 2021; Divya et al. 2019; Bohidar et al. 2024), 2 studies used the Wong‐Baker pain rating scale (Marques et al. 2023; Thakur et al. 2023), and 1 study used the Modified Wong‐Baker scale (Panchal, Jeevanandan, and Subramanian 2019). Unclear pain scale was reported in only 1 study (Moudgalya et al. 2024). Preoperative pain was clearly assessed in 4 papers (Thakur et al. 2023; Jeevanandan et al. 2020; Tyagi et al. 2021; Bohidar et al. 2024). Prescribed postoperative analgesics were unspecified in 7 studies (Marques et al. 2023; Hadwa et al. 2023; Barasuol et al. 2021; Tyagi et al. 2021; Divya et al. 2019; Moudgalya et al. 2024; Bohidar et al. 2024), whereas 4 studies (Thakur et al. 2023; Panchal, Jeevanandan, and Subramanian 2019; Jeevanandan et al. 2020; Topçuoğlu et al. 2017) prescribed ibuprofen or paracetamol as an alternative in case of hypersensitivity. None of the included studies clearly specified the dosages. The effect of postoperative analgesic intake on pain was evaluated in just 2 studies (Marques et al. 2023; Barasuol et al. 2021), without reporting any significant differences among assessed groups.

### 3.1 Risk of Bias

**Figure 2:**
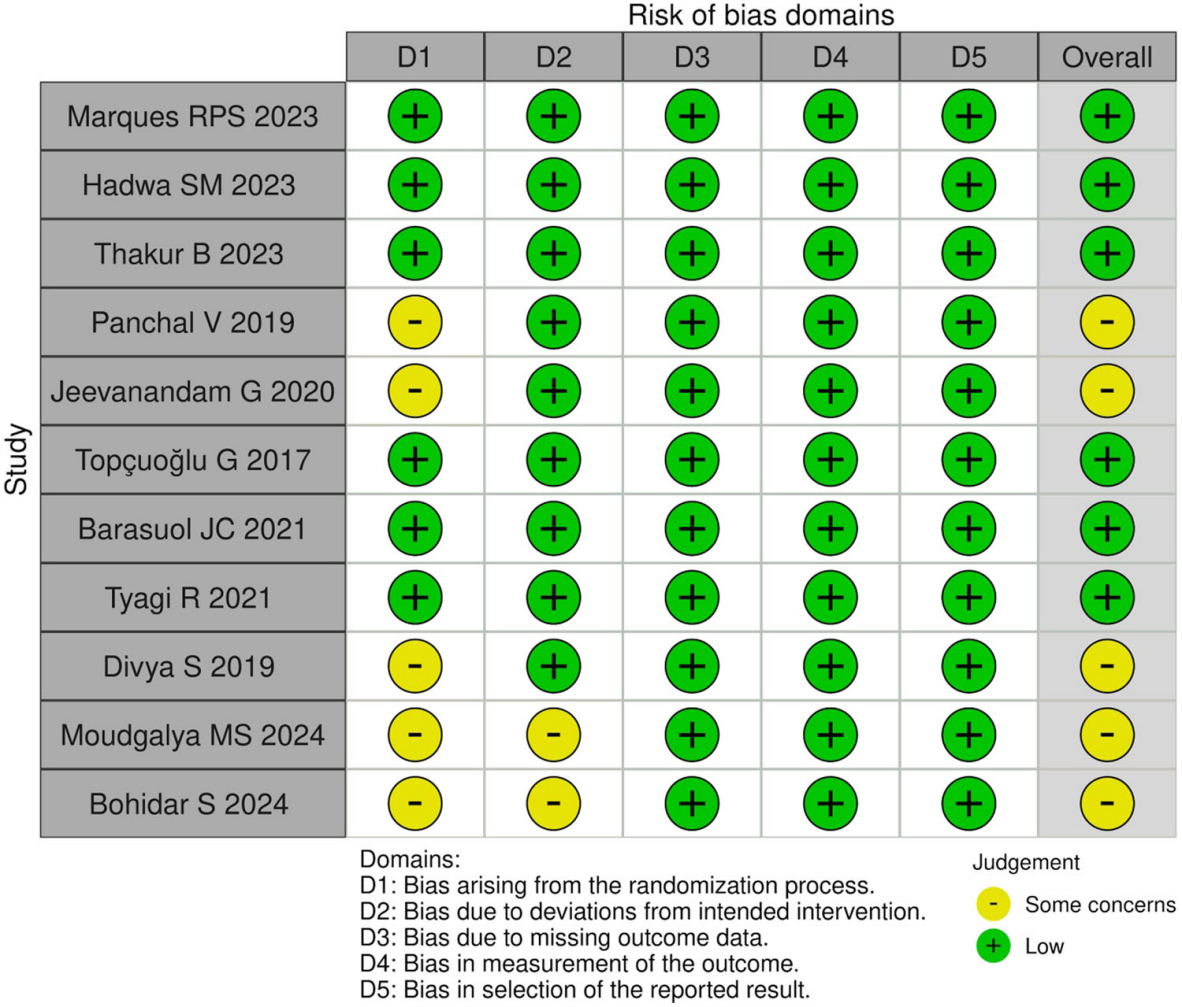
Risk of Bias Graph obtained by revised Cochrane risk‐of‐bias tool for randomized trials (RoB 2).

**Figure 3:**
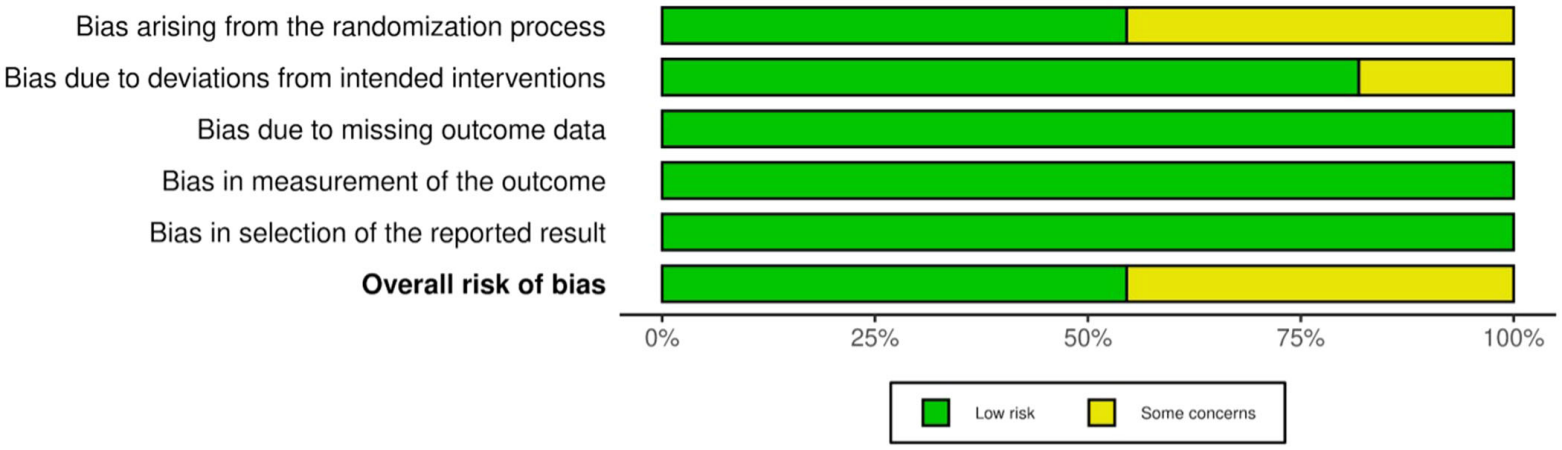
Risk of Bias Summary obtained by revised Cochrane risk‐of‐bias tool for randomized trials (RoB 2).

The quality of the included articles was assessed using the Cochrane Risk of Bias tool (RoB 2). Among the included studies, 5 demonstrated some concerns (Panchal, Jeevanandan, and Subramanian 2019; Jeevanandan et al. 2020; Divya et al. 2019; Moudgalya et al. 2024; Bohidar et al. 2024) while the remaining 6 had been categorized as having low risk of bias (Marques et al. 2023; Hadwa et al. 2023; Thakur et al. 2023; Topçuoğlu et al. 2017; Barasuol et al. 2021; Tyagi et al. 2021). The shortcomings mostly concerned the randomization process or deviations from the intended interventions (Figure 2). The overall risk of biased judgment of all studies was classified as low risk (Figure 3).

### 3.2 Meta‐Analysis

**Figure 4:**
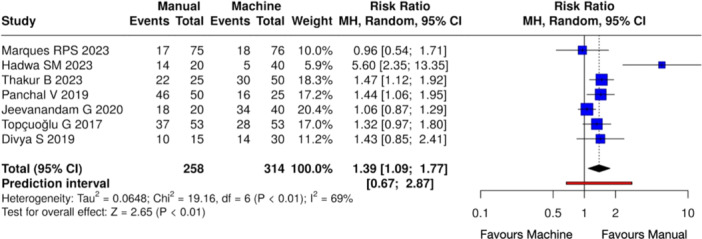
Forest plot of overall postoperative pain incidence between manual and mechanical instrumentation systems after pulpectomy procedures of primary teeth. Pain occurrence was statistically higher in the case of manual instrumentation.

Quantitative evaluation was performed on 7 studies (Marques et al. 2023; Hadwa et al. 2023; Thakur et al. 2023; Panchal, Jeevanandan, and Subramanian 2019; Jeevanandan et al. 2020; Topçuoğlu et al. 2017; Divya et al. 2019). Studies conducted by Barasuol et al. (2021) and Moudgalya et al. (2024) were excluded due to a lack of clarity in pain assessment during different time points. In addition, papers by Tyagi et al. (2021) and Bohidar et al. (2024) were excluded since pain scores were provided only as mean values and standard deviations. A random effects model was employed due to variations in patients' demographic data, initial pulpal diagnosis, irrigation protocol, instrumentation technique, and intracanal medicaments used. Follow‐up time periods, as well as the pain assessment scales, varied among the studies. A statistically significant difference in the overall pain incidence was observed between manual and mechanical instrumentation systems, with favorable outcomes for the latter after pulpectomy procedures (Heterogeneity: Tau² = 0.0648; Chi² = 19.16, *df *= 6 (*p* < 0.01); *I* ^2^ = 69%, *Z* = 2.65 (*p* < 0.01)) (Figure 4).

**Figure 5:**
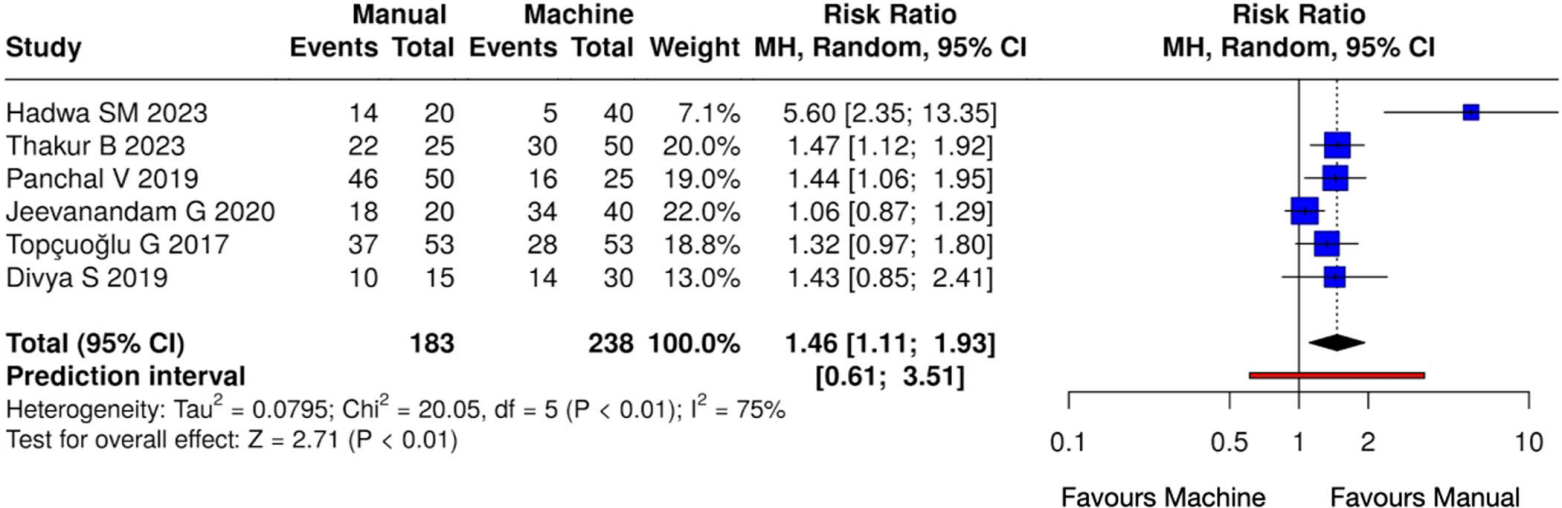
Forest plot of overall postoperative pain incidence at 6 h between manual and mechanical instrumentation systems after pulpectomy procedures of primary teeth. Pain occurrence was statistically higher in the case of manual instrumentation.

**Figure 6:**
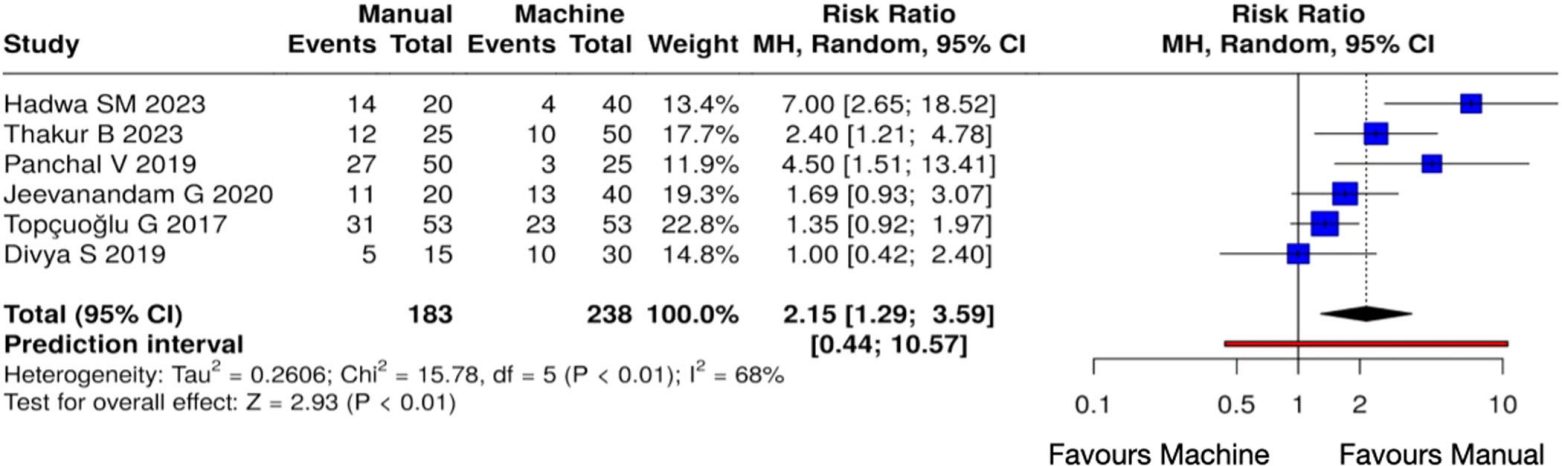
Forest plot of overall postoperative pain incidence at 12 h between manual and mechanical instrumentation systems after pulpectomy procedures of primary teeth. Pain occurrence was statistically higher in the case of manual instrumentation.

**Figure 7:**
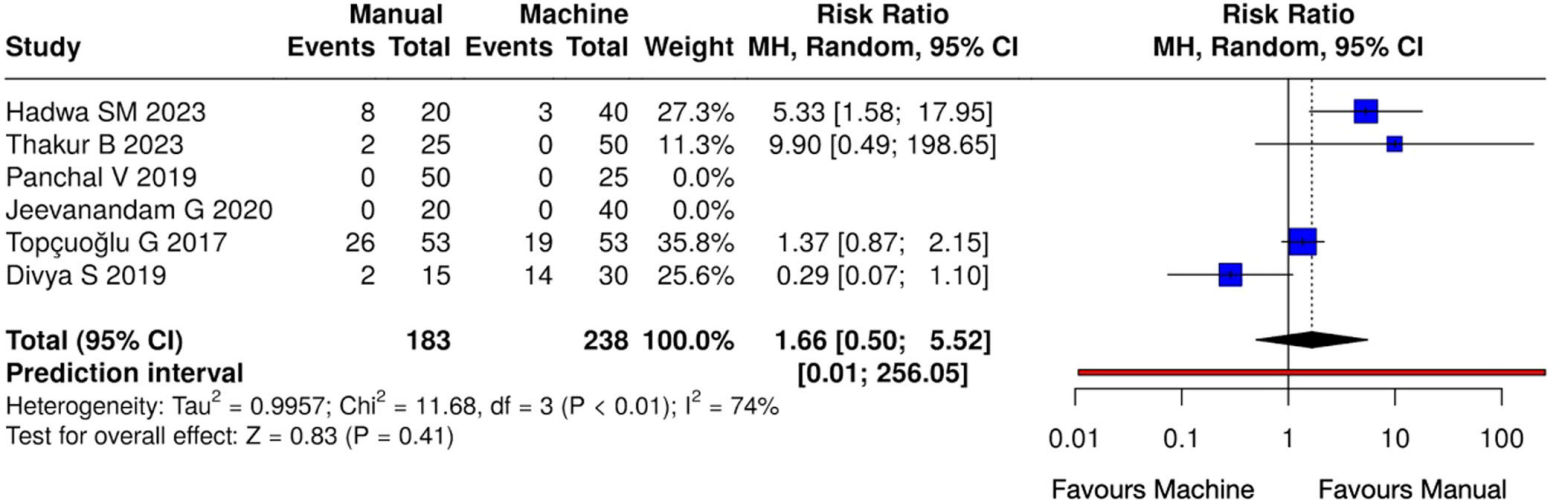
Forest plot of overall postoperative pain incidence at 24 h between manual and mechanical instrumentation systems after pulpectomy procedures of primary teeth. There was no statistically significant difference between manual and mechanical instrumentation.

**Figure 8:**
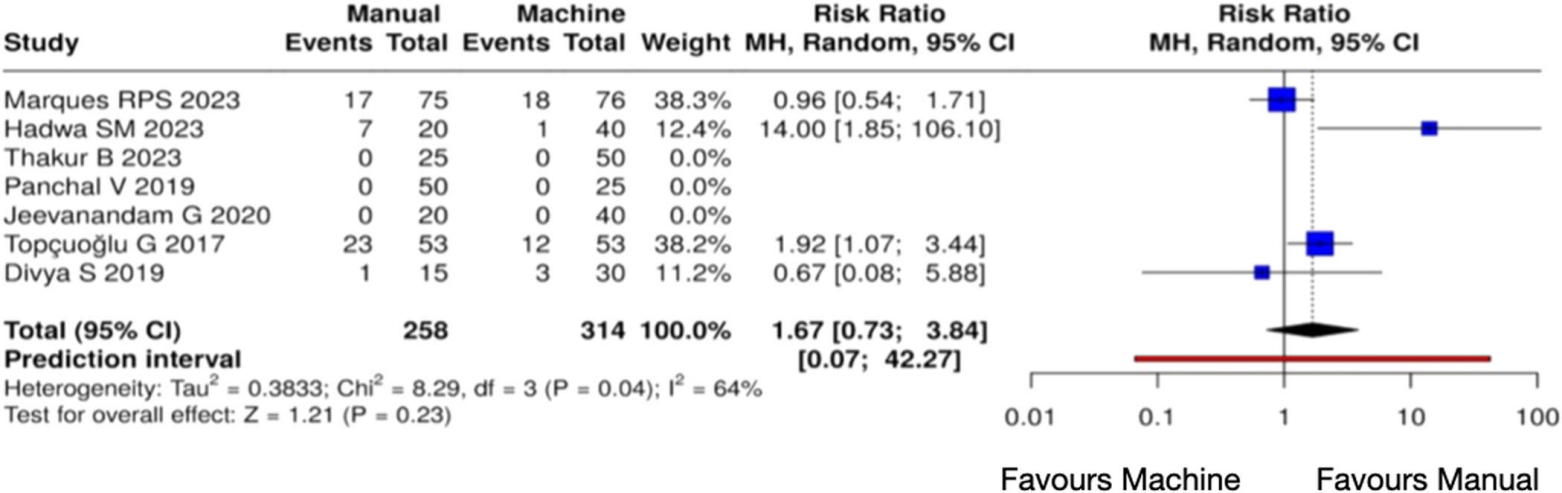
Forest plot of overall postoperative pain incidence at 48 h between manual and mechanical instrumentation systems after pulpectomy procedures of primary teeth. There was no statistically significant difference between manual and mechanical instrumentation.

**Figure 9:**
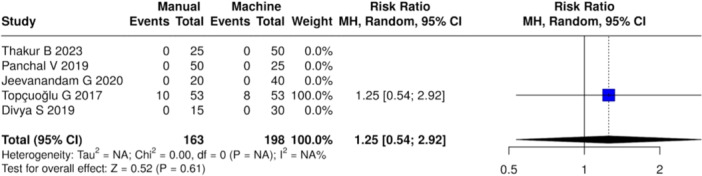
Forest plot of overall postoperative pain incidence at 72 h between manual and mechanical instrumentation systems after pulpectomy procedures of primary teeth. There was no statistically significant difference between manual and mechanical instrumentation.

Meta‐analyses at different time intervals showed a statistically significant difference in pain occurrence in the manual instrumentation groups at 6 h (Heterogeneity: Tau² = 0.0795; Chi² = 20.05, *df* = 5 (*p* < 0.01); *I* ^2^ = 75%, *Z* = 2.71 (*p* < 0.01)) (Figure 5) and 12 h (Heterogeneity: Tau² = 0.2606; Chi² = 15.78, *df* = 5 (*p* < 0.01); *I* ^2^ = 68%, *Z* = 2.93 (*p* < 0.01)) (Figure 6) when compared with mechanical shaping. On the other hand, no notable variations were observed between the manual and mechanical instrumentation groups at 24 h (Heterogeneity: Tau² = 0.9957; Chi² = 11.68, *df* = 3 (*p* < 0.01); *I* ^2^ = 74%, *Z* = 0.83 (*p* = 0.41)) (Figure 7), 48 h (Heterogeneity: Tau² = 0.3833; Chi² = 8.29, *df* = 3 (*p* = 0.04); *I* ^2^ = 64%, *Z* = 1.21 (*p* = 0.23)) (Figure 8), and 72 h (Heterogeneity: Tau² = NA; Chi² = 0.00, *df* = 0 (*p* = NA); *I* ^2^ = NA%, *Z* = 0.52 (*p* = 0.61)) (Figure 9).

## 4 Discussion

The current systematic review and meta‐analysis were conducted to evaluate the effectiveness of manual and mechanical root canal instrumentations in reducing postoperative pain incidence following pulpectomy procedures. Previous systematic reviews have analyzed various aspects of root canal treatment, including success rates, quality of root canal filling, instrumentation, cleaning efficacy and postoperative pain occurrence (Manchanda et al. 2020; Lakshmanan et al. 2021; Asokan et al. 2021; Padmawar et al. 2025; Chugh et al. 2021; Kalaskar et al. 2024).

Regarding pain incidence following the use of various instrumentation systems, a previous study (Lakshmanan et al. 2021) reported data only on 3 papers, while the present paper provided a comprehensive review analyzing 11 studies from a qualitative point of view and 7 studies were further quantitatively evaluated. In accordance with scientific literature, it's been demonstrated that rotary instrumentation was significantly superior to hand instrumentation in reducing overall postoperative pain. It should be considered that primary teeth are more predisposed to debris apical extrusion due to root anatomy as well as physiological root resorption; in this light, mechanical instrumentation seemed to reduce the amount of debris pushed into periapical tissue (Rahbani Nobar et al. 2021) and, thanks to better cutting abilities, decrease the instrumentation time, contributing to lesser pain incidence than manual techniques (Panchal, Jeevanandan, and Erulappan 2019; Khadmi et al. 2025). Although in permanent dentition differences of apical extrusion of debris caused by rotary and reciprocating systems are reported (Alhayki 2025; Villani et al. 2024; Western and Dicksit 2017), regarding primary teeth, the degree of extruded debris by different mechanical instrumentations should be better elucidated. A lesser extent of apical extrusion has been observed when using dedicated pediatric rotary systems (Kamatchi et al. 2024).

The analysis of root canals of primary teeth after instrumentation revealed a higher preservation of remaining dentin thickness caused by manual preparation and the production of less dentin cracks when compared to rotary and reciprocating systems (Saha and Singh 2022), even though iatrogenic errors such as canal transportation and apical blockage have been observed (George et al. 2016). On the other hand, the reciprocating systems significantly reduced the instrumentation time and the rotary ones produced lesser canal transportation (Saha and Singh 2022), obtaining a better canal shaping as well as tridimensional space for correct obturation (Kumaran et al. 2024). Limitations of mechanical instrumentation are commonly due to instrument fracture, overheating, and loss of hard tissue, mostly in the case of thinner root dentin. These drawbacks might be overcome by new thermal treatments and motions to increase mechanical performances, limit the risks of iatrogenic complications, and mitigate posttreatment pain incidence (Azizi and Azizi 2021; Pelliccioni et al. 2023; Tabassum et al. 2019; Zupanc et al. 2018).

The conducted meta‐analyses suggested a significant pain reduction in the case of mechanical instrumentation 6 and 12 h after endodontic treatment. On the other hand, no significant differences were observed at 24, 42, 72 h. As for permanent dentition, pain following root canal therapy is most commonly experienced 6 and 12 h after treatment (Smith et al. 2017), underlying how critical that time interval is. In addition, the present study considered an evaluation range until 72 h, that represented the interval time in which post‐endodontic pain is mainly represented (Shamszadeh et al. 2020). Pain is inherently multifactorial, and its subjective nature varies across individuals (Spohr et al. 2019). In addition, other variables might contribute to pain incidence and intensity, as correct use of irrigants, obturation technique, number of visits/interventions, and operator's skills (Ng et al. 2004; Valizadeh et al. 2024; Tirupathi et al. 2019; Priyadarshini 2021). Regarding disinfection protocols, some variations were reported by the included studies. 1% NaOCl was the most widely used concentration, followed by 2.5% and 3%. Moreover, EDTA or CHX were used in a few other studies, without precisely describing the volume or the irrigation regimen followed. These inconsistencies might be considered as confounding factors in postoperative pain incidence and should be taken into account in the interpretation of outcomes, although current data from high‐quality evidence revealed no significant differences between 1% NaOCl and higher concentrations in pulpectomy procedures (Gurunathan et al. 2024). Moreover, the effect on peri‐apical tissue of apical extrusion of irrigants or infected debris caused by irrigation procedures should be considered as additional cofounding variables in pain incidence (Valizadeh et al. 2024). Regarding obturation material, the iodoform‐calcium hydroxide combination was mostly used in the included studies, not representing a potential unfavorable factor in the interpretation of the results. Although extrusion of obturation material is an uncommon event in the treatment of primary teeth, the success of tridimensional sealing of the root canal system strictly depends on the proper technique of instrumentation (Priyadarshini 2021). All included studies, except for Moudgalya et al. (2024), performed pulpectomies in a single visit, without intracanal medication. This aspect might be relevant in the outcomes' interpretation and is in line with current scientific literature that emphasized the importance of completing the pulpectomy in a single visit whenever feasible (Tirupathi et al. 2019).

Quality analysis of included studies demonstrated an overall low risk of bias. All studies were randomized clinical trials, except for Bohidar et al. (2024) which was a clinical study with some concerns. Moreover, all included papers clearly reported their sample sizes, mainly employed 80% power for their calculations, and did not experience loss to follow‐up, reducing potential bias.

The results obtained by the present systematic review and meta‐analysis might provide a significant clinical implication, since in pediatric patients, treatment time is a crucial factor in reducing anxiety (Manchanda et al. 2020), which, in turn, improves operator efficiency and enhances therapeutic outcomes. Children often lack the communication skills or the ability to properly express discomfort, and together with the difficulty of the treatment procedure, as well as the need for rapid interventions, are of paramount importance in their management. In this regard, mechanical instrumentation is not only advantageous in decreasing overall treatment time, but also in reducing pain incidence after therapy. However, further clinical trials should be prospectively planned to support the obtained outcomes and precisely individualize all factors that would contribute to the clinical success over time, reducing side effects.

## 5 Limitations

Regarding the limitations of the current systematic review and meta‐analysis, it should be noted that the current review pooled together all types of instrumentation designs and kinematics, including rotary and reciprocating systems. Therefore, the extrapolation of results cannot be confined to a single system or design. Future systematic reviews are warranted to evaluate success rates and clinical outcomes, precisely assessing single instrumentation systems and making comparisons between systematics. In addition, the present study included papers dealing with non‐vital primary teeth or vital primary teeth with signs and symptoms of irreversible pulpitis, that were candidate to root canal therapy; however, the pulp status and degree of inflammation were impossible to be evaluated, and these aspects may have an impact in pain perception, healing and postoperative pain incidence. Moreover, the presence of preoperative pain was clearly assessed in only 4 papers (Thakur et al. 2023; Jeevanandan et al. 2020; Tyagi et al. 2021; Bohidar et al. 2024), and its impact on post‐endodontic pain remained difficult to figure out. Finally, it should be stressed that pain assessment was based on a subjective evaluation carried out by patients and/or their caregivers, expressed through different scales that assessed only pain presence/absence or its level as mild/moderate/severe. These aspects limited the clear description of pain without providing specific signs and symptoms.

## 6 Conclusion

The present systematic review and meta‐analysis demonstrated a statistically significant reduction in pain incidence 6 and 12 h after pulpectomy of primary teeth with mechanical instrumentation when compared to manual files. The obtained results might be useful not only in decreasing overall intervention time, but also in improving the therapeutic outcomes and ease pulpectomy that nowadays represents an important and widely used procedure to preserve primary teeth.

## Author Contributions

**Kavalipurapu Venkata Teja:** study design and data extraction. **Kaligotla Apoorva Vasundhara:** data extraction and meta‐analysis. **Gianrico Spagnuolo:** critical revision and results interpretation. **Niccolò Giuseppe Armogida:** artworks and literature search. **Flavia Iaculli:** writing and editing of the original manuscript. **Carlo Rengo:** supervision.

## Ethics Statement

The authors have nothing to report.

## Conflicts of Interest

The authors declare no conflicts of interest.

## Data Availability

The data that support the findings of this study are available from the corresponding author upon reasonable request.

## References

[cre270180-bib-0001] Ahmed, H. M. A. , P. K. Musale , O. I. El Shahawy , and P. M. H. Dummer . 2020. “Application of a New System for Classifying Tooth, Root and Canal Morphology in the Primary Dentition.” International Endodontic Journal 53: 27–35. 31390075 10.1111/iej.13199

[cre270180-bib-0002] Alhayki, M. M. 2025. “Evaluation of Apically Extruded Debris During Root Canal Preparation Using ProTaper Ultimate and ProTaper Gold: An Ex Vivo Study.” European Endodontic Journal 10: 41–46. 40145483 10.14744/eej.2024.43650 PMC11971712

[cre270180-bib-0003] Arias, A. , J. C. de la Macorra , J. J. Hidalgo , and M. Azabal . 2013. “Predictive Models of Pain Following Root Canal Treatment: A Prospective Clinical Study.” International Endodontic Journal 46: 784–793. 23402273 10.1111/iej.12059

[cre270180-bib-0004] Asokan, S. , N. Natchiyar , P. R. G. Priya , and T. D. Y. Kumar . 2021. “Comparison of Clinical and Radiographic Success of Rotary With Manual Instrumentation Techniques in Primary Teeth: A Systematic Review.” International Journal of Clinical Pediatric Dentistry 14: 8–13. 34326578 10.5005/jp-journals-10005-1879 PMC8311778

[cre270180-bib-0005] Azizi, A. , and A. Azizi . 2021. “In‐Depth Metallurgical and Microstructural Analysis of Oneshape and Heat Treated Onecurve Instruments.” European Endodontic Journal 6, no. 1: 90–97. 33762534 10.14744/eej.2020.63634 PMC8056813

[cre270180-bib-0006] Barasuol, J. C. , C. Massignan , E. A. Bortoluzzi , M. Cardoso , and M. Bolan . 2021. “Influence of Hand and Rotary Files for Endodontic Treatment of Primary Teeth on Immediate Outcomes: Secondary Analysis of a Randomized Controlled Trial.” International Journal of Paediatric Dentistry 31: 143–151. 32516507 10.1111/ipd.12682

[cre270180-bib-0007] Bohidar, S. , P. Goswami , A. Arya , S. Singh , P. V. Samir , and T. Bhargava . 2024. “An In Vivo Evaluation of Postoperative Pain After Root Canal Instrumentation Using Manual K‐Files and Kedo‐S Rotary Files in Primary Molars.” Journal of Pharmacy and BioAllied Sciences 16: S136–S139. 38595458 10.4103/jpbs.jpbs_420_23 PMC11001094

[cre270180-bib-0008] Chugh, V. K. , A. K. Patnana , A. Chugh , P. Kumar , P. Wadhwa , and S. Singh . 2021. “Clinical Differences of Hand and Rotary Instrumentations During Biomechanical Preparation in Primary Teeth – A Systematic Review and Meta‐Analysis.” International Journal of Paediatric Dentistry 31: 131–142. 32815216 10.1111/ipd.12720

[cre270180-bib-0009] Cleghorn, B. M. , N. B. Boorberg , and W. H. Christie . 2010. “Primary Human Teeth and Their Root Canal Systems.” Endodontic Topics 23: 6–33.

[cre270180-bib-0010] Divya, S. , G. Jeevanandan , S. Sujatha , E. G. Subramanian , and V. Ravindran . 2019. “Comparison of Quality of Obturation and Post‐Operative Pain Using Manual vs Rotary Files in Primary Teeth – A Randomised Clinical Trial.” Indian Journal of Dental Research 30: 904–908. 31939369 10.4103/ijdr.IJDR_37_18

[cre270180-bib-0011] George, S. , S. Anandaraj , J. S. Issac , S. A. John , and A. Harris . 2016. “Rotary Endodontics in Primary Teeth – A Review.” Saudi Dental Journal 28: 12–17. 26792964 10.1016/j.sdentj.2015.08.004 PMC4688451

[cre270180-bib-0012] Girish Babu, K. L. , K. Gururaj Hebbar , and G. M. Doddamani . 2024. “Correlation Between Quality of Obturation and Outcome of Pulpectomized Primary Molars Following Root Canal Instrumentation With Pediatric Rotary File Systems.” Pediatric Dental Journal 34: 27–34.

[cre270180-bib-0013] Gurunathan, D. , L. Thangavelu , and D. Mukundan . 2024. “Comparative Evaluation of 1% Sodium Hypochlorite vs Other Intracanal Irrigants During Pulpectomy of Primary Teeth: A Systematic Review.” World Journal of Dentistry 15: 451–456.

[cre270180-bib-0014] Hadwa, S. M. , R. F. Ghouraba , I. A. Kabbash , and S. S. El‐Desouky . 2023. “Assessment of Clinical and Radiographic Efficiency of Manual and Pediatric Rotary File Systems in Primary Root Canal Preparation: A Randomized Controlled Clinical Trial.” BMC Oral Health 23: 687. 37742023 10.1186/s12903-023-03393-1 PMC10518081

[cre270180-bib-0015] Jeevanandan, G. , V. Ravindran , E. M. Subramanian , and A. S. Kumar . 2020. “Postoperative Pain With Hand, Reciprocating, and Rotary Instrumentation Techniques After Root Canal Preparation in Primary Molars: A Randomized Clinical Trial.” International Journal of Clinical Pediatric Dentistry 13: 21–26. 32581473 10.5005/jp-journals-10005-1709 PMC7299878

[cre270180-bib-0016] Kalaskar, R. , V. Vinay , U. P. Gala , S. Joshi , and A. R. Doiphode . 2024. “Comparative Evaluation of Effectiveness of Rotary and Hand File Systems in Terms of Quality of Obturation and Instrumentation Time Among Primary Teeth: A Systematic Review and Meta‐Analysis of Randomized Controlled Trials.” International Journal of Clinical Pediatric Dentistry 17: 962–969. 39372345 10.5005/jp-journals-10005-2950 PMC11451864

[cre270180-bib-0017] Kamatchi, M. , M. Gawthaman , S. Vinodh , M. Manoharan , S. Kowsalya , and V. M. Mathian . 2024. “Comparative Evaluation of Apical Debris Extrusion in Primary Molars Using Three Different Pediatric Rotary Systems: An In Vitro Study.” International Journal of Clinical Pediatric Dentistry 17: 1224–1228. 39781393 10.5005/jp-journals-10005-2989 PMC11703772

[cre270180-bib-0018] Khadmi, I. , A. Hamrouni , and F. Chouchene . 2025. “Different Outcomes of Rotary and Manual Instrumentation in Primary Teeth Pulpectomy: A Systematic Review and Meta‐Analysis.” European Archives of Paediatric Dentistry 26: 423–450. 10.1007/s40368-025-01020-x. 40128471

[cre270180-bib-0019] Kumaran, P. , A. M. Xavier , M. Venugopal , et al. 2024. “Volumetric Analysis of Hand and Rotary Instrumentation, Root Canal Filling Techniques, and Obturation Materials in Primary Teeth Using Spiral CT.” Journal of Contemporary Dental Practice 25: 250–259. 38690699 10.5005/jp-journals-10024-3644

[cre270180-bib-0020] Lakshmanan, L. , S. Somasundaram , G. Jeevanandan , and E. Subramanian . 2021. “Evaluation of Postoperative Pain After Pulpectomy Using Different File Systems in Primary Teeth: A Systematic Review.” Contemporary Clinical Dentistry 12: 3–8. 33967530 10.4103/ccd.ccd_561_20 PMC8092099

[cre270180-bib-0021] Li, L. , X. Yang , W. Ju , J. Li , and X. Yang . 2023. “Impact of Primary Molars With Periapical Disease on Permanent Successors: A Retrospective Radiographic Study.” Heliyon 9: e15854. 37187910 10.1016/j.heliyon.2023.e15854 PMC10176069

[cre270180-bib-0022] Manchanda, S. , D. Sardana , and C. K. Y. Yiu . 2020. “A Systematic Review and Meta‐Analysis of Randomized Clinical Trials Comparing Rotary Canal Instrumentation Techniques With Manual Instrumentation Techniques in Primary Teeth.” International Endodontic Journal 53: 333–353. 31587323 10.1111/iej.13233

[cre270180-bib-0023] Marques, R. P. S. , N. M. Oliveira , V. R. P. Barbosa , et al. 2023. “Reciprocating Instrumentation for Endodontic Treatment of Primary Molars: 24‐month Randomized Clinical Trial.” International Journal of Paediatric Dentistry 33: 325–334. 36522131 10.1111/ipd.13042

[cre270180-bib-0024] Martins, C. , V. De Souza Batista , A. Andolfatto Souza , A. Andrada , G. Mori , and J. Gomes Filho . 2019. “Reciprocating Kinematics Leads to Lower Incidences of Postoperative Pain Than Rotary Kinematics After Endodontic Treatment: A Systematic Review and Meta‐Analysis of Randomized Controlled Trial.” Journal of Conservative Dentistry 22: 320–331. 31802813 10.4103/JCD.JCD_439_18 PMC6873607

[cre270180-bib-0025] Morankar, R. , A. Goyal , K. Gauba , A. Kapur , and S. K. Bhatia . 2018. “Manual Versus Rotary Instrumentation for Primary Molar Pulpectomies – A 24 Months Randomized Clinical Trial.” Pediatric Dental Journal 28: 96–102.

[cre270180-bib-0026] Moudgalya, M. S. , P. Tyagi , S. Tiwari , T. Tiwari , P. Umarekar , and S. Shrivastava . 2024. “To Compare and Evaluate Rotary and Manual Techniques in Biomechanical Preparation of Primary Molars to Know Their Effects in Terms of Cleaning and Shaping Efficacy.” International Journal of Clinical Pediatric Dentistry 17: 864–870. 39372346 10.5005/jp-journals-10005-2949 PMC11451875

[cre270180-bib-0027] Ng, Y. L. , J. P. Glennon , D. J. Setchell , and K. Gulabivala . 2004. “Prevalence of and Factors Affecting Post‐Obturation Pain in Patients Undergoing Root Canal Treatment.” International Endodontic Journal 37: 381–391. 15186245 10.1111/j.1365-2591.2004.00820.x

[cre270180-bib-0028] Nisar, P. , F. Katge , V. K. Chimata , D. Pradhan , D. Patil , and I. Agrawal . 2024. “Comparative Evaluation of Hand and Rotary File Systems on Dentinal Microcrack Formation During Pulpectomy Procedure in Primary Teeth: An In Vitro Study.” European Archives of Paediatric Dentistry 25: 181–189. 38461490 10.1007/s40368-024-00863-0

[cre270180-bib-0029] Padmawar, N. , N. Pawar , V. Tripathi , S. Banerjee , G. Tyagi , and S. R. Joshi . 2025. “Comparative Analysis of Rotary Versus Manual Instrumentation in Paediatric Pulpectomy Procedures: A Systematic Review and Meta‐Analysis.” Australian Endodontic Journal 51: 181–196. 10.1111/aej.12899. 39494967

[cre270180-bib-0030] Page, M. J. , J. E. McKenzie , P. M. Bossuyt , et al. 2021. “The PRISMA 2020 Statement: An Updated Guideline for Reporting Systematic Reviews.” BMJ 372: n71. 33782057 10.1136/bmj.n71 PMC8005924

[cre270180-bib-0031] Panchal, V. , G. Jeevanandan , and S. M. Erulappan . 2019. “Comparison Between the Effectiveness of Rotary and Manual Instrumentation in Primary Teeth: A Systematic Review.” International Journal of Clinical Pediatric Dentistry 12: 340–346. 31866721 10.5005/jp-journals-10005-1637 PMC6898866

[cre270180-bib-0032] Panchal, V. , G. Jeevanandan , and E. M. G. Subramanian . 2019. “Comparison of Post‐Operative Pain After Root Canal Instrumentation With Hand K‐Files, H‐Files and Rotary Kedo‐S Files in Primary Teeth: A Randomised Clinical Trial.” European Archives of Paediatric Dentistry 20: 467–472. 30864090 10.1007/s40368-019-00429-5

[cre270180-bib-0033] Pelliccioni, G. A. , R. Schiavon , F. Zamparini , et al. 2023. “Physico‐Mechanical Properties of Two Different Heat Treated Nickel‐Titanium Instruments: In Vitro Study.” Giornale Italiano Di Endodonzia 38, no. 1: 28–36.

[cre270180-bib-0034] Priyadarshini, P. 2021. “Comparative Evaluation of Quality of Obturation and Its Effect on Postoperative Pain Between Pediatric Hand and Rotary Files: A Double‐Blinded Randomized Controlled Trial.” International Journal of Clinical Pediatric Dentistry 14: 88–96. 34326591 10.5005/jp-journals-10005-1895 PMC8311770

[cre270180-bib-0035] Rahbani Nobar, B. , O. Dianat , B. Rahbani Nobar , et al. 2021. “Effect of Rotary and Reciprocating Instrumentation Motions on Postoperative Pain Incidence in Non‐Surgical Endodontic Treatments: A Systematic Review and Meta‐Analysis.” European Endodontic Journal 6: 3–14. 33609019 10.14744/eej.2020.51523 PMC8056811

[cre270180-bib-0036] Saha, S. , and P. Singh . 2022. “Cone‐Beam Computed Tomographic Analysis of Deciduous Root Canals After Instrumentation With Different Filing Systems: An In Vitro Study.” International Journal of Clinical Pediatric Dentistry 15: S22–S29. 35645508 10.5005/jp-journals-10005-2126 PMC9108822

[cre270180-bib-0037] Schachter, D. , S. Blumer , S. Sarsur , et al. 2023. “Exploring a Paradigm Shift in Primary Teeth Root Canal Preparation: An Ex Vivo Micro‐CT Study.” Children 10: 792. 37238340 10.3390/children10050792 PMC10217407

[cre270180-bib-0038] Shamszadeh, S. , A. Shirvani , and S. Asgary . 2020. “Does Occlusal Reduction Reduce Post‐Endodontic Pain? A Systematic Review and Meta‐Analysis.” Journal of Oral Rehabilitation 47: 528–535. 31880822 10.1111/joor.12929

[cre270180-bib-0039] Shetty, B. , R. Singh , V. Patil , S. P. Tirupathi , K. Nene , and N. Rathi . 2023. “Comparative Evaluation of Single Rotary File System and Sequential Multi‐File Rotary Systems on Time for Biomechanical Preparation and Obturation Quality in Single‐Visit Pulpectomy Protocol: A Double‐Blind Randomized Clinical Trial.” International Journal of Clinical Pediatric Dentistry 16: 247–252. 38268640 10.5005/jp-journals-10005-2685 PMC10804288

[cre270180-bib-0040] Smith, E. A. , J. G. Marshall , S. S. Selph , D. R. Barker , and C. M. Sedgley . 2017. “Nonsteroidal Anti‐Inflammatory Drugs for Managing Postoperative Endodontic Pain in Patients Who Present With Preoperative Pain: A Systematic Review and Meta‐Analysis.” Journal of Endodontics 43: 7–15. 27939729 10.1016/j.joen.2016.09.010

[cre270180-bib-0041] Spohr, A. R. , R. Sarkis‐Onofre , T. Pereira‐Cenci , F. G. Pappen , and R. Dornelles Morgental . 2019. “A Systematic Review: Effect of Hand, Rotary and Reciprocating Instrumentation on Endodontic Postoperative Pain.” Giornale Italiano Di Endodonzia 33: 24–34.

[cre270180-bib-0042] Sterne, J. A. C. , J. Savović , M. J. Page , et al. 2019. “RoB 2: A Revised Tool for Assessing Risk of Bias in Randomised Trials.” BMJ 366: l4898. 31462531 10.1136/bmj.l4898

[cre270180-bib-0043] Sun, C. , J. Sun , M. Tan , B. Hu , X. Gao , and J. Song . 2018. “Pain After Root Canal Treatment With Different Instruments: A Systematic Review and Meta‐Analysis.” Oral Diseases 24: 908–919. 29516592 10.1111/odi.12854

[cre270180-bib-0044] Suresh, B. , G. Jeevanandan , V. Ravindran , et al. 2023. “Comparative Evaluation of Extrusion of Apical Debris in Primary Maxillary Anterior Teeth Using Two Different Rotary Systems and Hand Files: An In Vitro Study.” Children 10: 898. 37238446 10.3390/children10050898 PMC10217004

[cre270180-bib-0045] Tabassum, S. , K. Zafar , and F. Umer . 2019. “Nickel‐Titanium Rotary File Systems: What's New?” European Endodontic Journal 4: 111–117. 32161896 10.14744/eej.2019.80664 PMC7006588

[cre270180-bib-0046] Thakur, B. , A. Bhardwaj , D. A. Wahjuningrum , et al. 2023. “Incidence of Post‐Operative Pain Following a Single‐Visit Pulpectomy in Primary Molars Employing Adaptive, Rotary, and Manual Instrumentation: A Randomized Clinical Trial.” Medicina 59: 355. 36837556 10.3390/medicina59020355 PMC9966361

[cre270180-bib-0047] Tirupathi, S. P. , N. Krishna , S. Rajasekhar , and S. Nuvvula . 2019. “Clinical Efficacy of Single‐Visit Pulpectomy Over Multiple‐Visit Pulpectomy in Primary Teeth: A Systematic Review.” International Journal of Clinical Pediatric Dentistry 12: 453–459. 32440053 10.5005/jp-journals-10005-1654 PMC7229364

[cre270180-bib-0048] Topçuoğlu, G. , H. S. Topçuoğlu , E. Delikan , M. Aydınbelge , and S. Dogan . 2017. “Postoperative Pain After Root Canal Preparation With Hand and Rotary Files in Primary Molar Teeth.” Pediatric Dentistry 39: 192–196. 28583242

[cre270180-bib-0049] Tyagi, R. , A. Khatri , N. Kalra , and P. Sabherwal . 2021. “Comparative Evaluation of Hand K‐Flex Files, Pediatric Rotary Files, and Reciprocating Files on Instrumentation Time, Postoperative Pain, and Child's Behavior in 4–8‐Year‐Old Children.” International Journal of Clinical Pediatric Dentistry 14: 201–206. 34413592 10.5005/jp-journals-10005-1919 PMC8343677

[cre270180-bib-0050] Valizadeh, M. , A. Gheidari , N. Daghestani , Z. Mohammadzadeh , and F. Khorakian . 2024. “Evaluation of Various Root Canal Irrigation Methods in Primary Teeth: A Systematic Review.” BMC Oral Health 24: 1535. 39709405 10.1186/s12903-024-05164-y PMC11662475

[cre270180-bib-0051] Villani, F. A. , F. Zamparini , A. Spinelli , R. Aiuto , and C. Prati . 2024. “Apical Debris Extrusion and Potential Risk of Endodontic Flare‐Up: Correlation With Rotating and Reciprocating Instruments Used in Daily Clinical Practice.” Giornale Italiano Di Endodonzia 38, no. 1: 79–23.

[cre270180-bib-0052] Western, J. , and D. Dicksit . 2017. “Apical Extrusion of Debris in Four Different Endodontic Instrumentation Systems: A Meta‐Analysis.” Journal of Conservative Dentistry 20: 30–36. 28761250 10.4103/0972-0707.209066 PMC5514807

[cre270180-bib-0053] Zupanc, J. , N. Vahdat‐Pajouh , and E. Schäfer . 2018. “New Thermomechanically Treated NiTi Alloys – A Review.” International Endodontic Journal 51: 1088–1103. 29574784 10.1111/iej.12924

